# Supplementary material for: COZOID: contact zone identifier for visual analysis of protein-protein interactions
Source: BMC Bioinformatics. 2018 Apr 6;19:125. doi: 10.1186/s12859-018-2113-6 (PMC5889581; doi:10.1186/s12859-018-2113-6)
Supplement: Supplementary file 2 — Software build. Executable binary file of the software tool. (ZIP 118,784 kb) [file 12859_2018_2113_MOESM2_ESM.zip › cozoid/java/docs/beansbinding-1.2.1-doc.zip!/org/jdesktop/el/FunctionMapper.html]

FunctionMapper (Beans Binding)


---


|  |  |  |
| --- | --- | --- |
| **Overview** \| **Package** \| **Class** \| **Use** \| **Tree** \| **Deprecated** \| **Index** \| **Help** | |  |
| **PREV CLASS**   **NEXT CLASS** | **FRAMES**    **NO FRAMES**     **All Classes** |  |
| SUMMARY: NESTED \| FIELD \| CONSTR \| METHOD | DETAIL: FIELD \| CONSTR \| METHOD |  |


---


## org.jdesktop.el Class FunctionMapper

```
java.lang.Object
  org.jdesktop.el.FunctionMapper
```

**Direct Known Subclasses:**: FunctionMapperFactory, FunctionMapperImpl

---

``` public abstract class FunctionMapper extends java.lang.Object ```

The interface to a map between EL function names and methods.

A `FunctionMapper` maps `${prefix:name()}`
style functions to a static method that can execute that function.

**Since:**
:   JSP 2.1

---

**Constructor Summary**

`FunctionMapper()`


| **Method Summary** | |
| --- | --- |
| `abstract  java.lang.reflect.Method` | `resolveFunction(java.lang.String prefix, java.lang.String localName)`             Resolves the specified prefix and local name into a `java.lang.Method`. |

**Methods inherited from class java.lang.Object**

`clone, equals, finalize, getClass, hashCode, notify, notifyAll, toString, wait, wait, wait`

**Constructor Detail**

### FunctionMapper

```
public FunctionMapper()
```


**Method Detail**

### resolveFunction

```
public abstract java.lang.reflect.Method resolveFunction(java.lang.String prefix,
                                                         java.lang.String localName)
```

:   Resolves the specified prefix and local name into a
    `java.lang.Method`.

    Returns `null` if no function could be found that matches
    the given prefix and local name.

    :   **Parameters:**: `prefix` - the prefix of the function, or "" if no prefix. For example, `"fn"` in `${fn:method()}`, or `""` in `${method()}`.: `localName` - the short name of the function. For example, `"method"` in `${fn:method()}`. **Returns:**: the static method to invoke, or `null` if no match was found.


---


|  |  |  |
| --- | --- | --- |
| **Overview** \| **Package** \| **Class** \| **Use** \| **Tree** \| **Deprecated** \| **Index** \| **Help** | |  |
| **PREV CLASS**   **NEXT CLASS** | **FRAMES**    **NO FRAMES**     **All Classes** |  |
| SUMMARY: NESTED \| FIELD \| CONSTR \| METHOD | DETAIL: FIELD \| CONSTR \| METHOD |  |


---
